# Risk factors for cardiopulmonary and respiratory arrest in medical and surgical hospital patients on opioid analgesics and sedatives

**DOI:** 10.1371/journal.pone.0194553

**Published:** 2018-03-22

**Authors:** Igor Izrailtyan, Jiejing Qiu, Frank J. Overdyk, Mary Erslon, Tong J. Gan

**Affiliations:** 1 Department of Anesthesiology, Stony Brook University, Stony Brook, NY, United States of America; 2 Health Economics and Outcomes Research, Medtronic, Mansfield, MA, United States of America; 3 Department of Anesthesiology, Roper St Francis Health System, Charleston, SC, United States of America; 4 Respiratory, Gastrointestinal & Informatics, Medtronic, Boulder, CO, United States of America; Harvard Medical School, UNITED STATES

## Abstract

**Background:**

Opioid induced respiratory depression is a known cause of preventable death in hospitals. Medications with sedative properties additionally potentiate opioid-induced respiratory and sedative effects, thereby elevating the risk for adverse events. The goal of this study was to determine what specific factors increase the risk of in-hospital cardiopulmonary and respiratory arrest (CPRA) in medical and surgical patients on opioid and sedative therapy.

**Methods:**

The present study analyzed 14,504,809 medical inpatient and 6,771,882 surgical inpatient discharges reported into the Premier database from 2008 to 2012. Patients were divided in four categories: on opioids; on sedatives; on both opioids and sedatives; and on neither opioids nor sedatives.

**Results:**

During hospital admission, 57% of all medical patients and 90% of all surgical patients were prescribed opioids, sedatives, or both. Surgical patients had a higher incidence of CPRA than medical patients (6.17 vs. 3.77 events per 1000 admissions; Relative Risk: 1.64 [95%CI: 1.62–1.66; *p*<0.0001). Opioids and sedatives were found to be independent predictors of CPRA (adjusted OR of 2.24 [95%CI: 2.18–2.29] for opioids and adjusted OR 1.80 [95%CI: 1.75–1.85] for sedatives in medical patients, and adjusted OR of 1.12 [95%CI: 1.07–1.16] for opioids and adjusted OR of 1.58 [95%CI: 1.51–1.66] for sedatives in surgical patients), with the highest risk in groups who received both types of medications (adjusted OR of 3.83 [95% CI: 3.74–3.92] in medical patients, and adjusted OR of 2.34 [95% CI: 2.25–2.42] in surgical patients) compared with groups that received neither type of medication. The common risk factors of CPRA in medical and surgical patients receiving both opioids and sedatives were Hispanic origin, mild liver disease, obesity, and COPD. Additionally, medical and surgical groups had their own unique risk factors for CPRA when placed on opioid and sedative therapy.

**Conclusions:**

Opioids and sedatives are independent and additive predictors of CPRA in both medical and surgical patients. Receiving both classes of medications further exacerbates the risk of CPRA for these patients. By identifying groups at risk among medical and surgical in-hospital patients, this study provides a step towards improving our understanding of how to use opioid and sedative medications safely, which may influence our treatment strategies and outcomes. More precise monitoring of selected high-risk patients may help prevent catastrophic cardiorespiratory complications from these medications. As a retrospective administrative database analysis, this study does not establish the causality or the temporality of the events but rather draws statistically significant associations between the clinical factors and outcomes.

## Introduction

Cardiopulmonary arrest remains a prominent public health burden in developed countries [1, 2]. With over half a million cardiac arrests occurring in the United States each year, over 200,000 of them take place in hospitals [1, 3]. While both the in-hospital cardiac arrest (IHCA) and out-of hospital cardiac arrest (OHCA) share similarities, they are very different entities [4–6]. In an OHCA, patients typically have a primary cardiac cause and an unexpected development of the arrest. Hospital patients who experience IHCA, however, usually have significant comorbidities and are characterized by predictable pathophysiologic changes manifesting critical illness [4–6]. Recognizing the different challenges faced by primary providers for IHCA and OHCA events, the 2015 American Heart Association (AHA) Guidelines for Cardiopulmonary Resuscitation and Emergency Cardiovascular Care have emphasized the need for two distinct pathways of care required to manage these two patient populations [4]. As the first link in the sequence of interventions in the chain of survival for patients with IHCA, AHA introduced a step calling for the surveillance and prevention of cardiac arrest in the hospital in order to facilitate a timely response and interventions.

Opioid-induced respiratory depression (OIRD), as a complication of a common approach to pain control management, is one of the major causes of preventable adverse events in the hospital [7–10]. According to a recent Anesthesia Closed Claims Project study [8], as much as 97% of all claims related to OIRD could probably or possibly be prevented. Benzodiazepines and other sedative medications can further increase the risk of adverse events in conjunction with opioids in both in-hospital and out-of-hospital settings [7, 8, 11–13]. According to the US Centers for Disease Control and Prevention, benzodiazepines were involved in 31% of opioid-analgesic poisoning deaths [12]. Over the past decade and in the more recent era of opioid abuse epidemic, there has been an upward trend in the presence of benzodiazepines in opioid poisoning [12].

If left unrecognized, OIRD is a life-threatening complication, which can lead to respiratory arrest or cardiopulmonary arrest [8–11]. While respiratory-only events may not necessarily degrade into cardiopulmonary events, it can be difficult to characterize the precipitating event(s) for cardiac events. In a recent study of over 21 million inpatient discharges reported in the Premier database, these life-threatening cardiopulmonary or respiratory arrest (CPRA) events were considered as a common entity expressing the most severe outcome in inpatients receiving opioids and sedatives [14]. In this prior analysis, opioids and sedatives were shown to be associated with an independent and additive risk of developing in-hospital CPRA [14]. Given that both medical and surgical patient populations are at risk for developing OIRD in hospital settings [7, 8, 15], the present study, using the same dataset, sought to identify specific clinical, demographic, and hospital-related risk factors leading to CPRA in medical and surgical patients on opioid and sedative therapy. This new and separate analysis presented in the current study will further help identify preventive strategies in specific patient populations [16] that are at risk for developing this devastating in-hospital complication.

## Methods

### Data source

Inpatient hospitalization billing data devoid of protected health information from Premier Inc. (Charlotte, North Carolina) was analyzed retrospectively in this study, exempting approval from an Institutional Review Board. The Premier database is one of the largest hospital-level resource utilization databases in the U.S., and represents approximately 1 out of every 5 U.S. inpatient hospitalizations from a diverse group of more than 600 hospitals. Each hospital submits quarterly data updates, which undergo rigorous validation checks and reconciliation. Each set of discharge-level data includes de-identified information about patient and provider characteristics, International Classification of Diseases 9th revision Clinical Modification (ICD-9-CM) diagnosis and procedure codes, Current Procedural Terminology (CPT), Disease Related Group (DRG), admission type, length of stay, discharge status, hospital resource utilization and cost of care [17].

### Patient population

All inpatient discharges reported to the Premier database between January 2008 and December 2012 were identified. Patients that were 18-years-old or younger, had CPRA on admission, came in with existing acute respiratory failure or neuromuscular disorder (see S1 Table), or lacked medical or surgical information, were excluded. The final dataset included 21,276,691 patients. CPRA cases were identified using the ICD-9-CM diagnosis codes of 427.5 (cardiopulmonary arrest) and 799.1 (respiratory arrest) and the ICD-9 procedure code of 99.60 or CPT code of 92950 (cardiopulmonary resuscitation). All pharmacy billing information was extracted to identify respiratory depressant medications, and then classified into four mutually exclusive categories: (1) opioids only, (2) sedatives only, (3) both opioids and sedatives, and (4) neither opioids nor sedatives. Supporting Information (see S2 Table) provides the complete list of medications.

### Statistical analysis

Descriptive statistics were constructed with the use of frequencies and proportions for categorical data and means and standard deviations (SD) for continuous variables. Categorical variables were compared using the chi-square test or Fisher Exact test, whereas the continuous variables were compared using a two-tailed student’s t-test. Variables of patient and hospital characteristics which were available through Premier databases, along with all comorbidities defined by the Charlson Comorbidity Index (CCI) such as myocardial infarction, congestive heart failure, dementia, chronic obstructive pulmonary disease (COPD), rheumatoid arthritis, peptic ulcer disease, paralysis, chronic renal failure, cancer, metastatic solid tumor, AIDS, obesity, diabetes, hypertension, peripheral vascular disease, cardiovascular disease, mild liver disease and moderate-severe liver disease were selected for a multivariable logistic regression model. Final variables for the multivariable model included: opioid and sedative administration, patient information such as age, gender, race, comorbid conditions described above, history of opioid usage, sleep disorder, smoking, admission type (elective or emergency), and hospital characteristics including region, bed size, rural vs. urban, and teaching vs. non-teaching. The medical and surgical patients with and without opioids and sedatives were analyzed separately. The adjusted odds ratio (OR) for each level of covariate was estimated by multivariable logistic regression model and c-statistic was used to evaluate the model’s predictive accuracy. All analyses were performed using SAS version 9.2 (SAS Institute Inc., Cary, NC). Results with a *p*-value of less than 0.05 were considered statistically significant.

## Results

### Incidence of CPRA

Among 14,504,809 medical patient and 6,771,882 surgical patient discharges, there were a total of 96,554 CPRA events (see Table 1). The CPRA rate per admission was almost two times higher for surgical patients as compared to medical patients (6.17 vs. 3.77 events per 1000 admissions; Relative Risk: 1.64 [95%CI: 1.62–1.66; *p*<0.0001). Among all hospital deaths, 25.7% vs. 14.4% were preceded by CPRA in the surgical vs. medical patient population, respectively. During hospital admission, 57% of all medical patients (8,251,842 patients) were placed on either sedative or opioid medications. The number of surgical patients managed with either sedatives or opioids reached 90% (6,105,167 surgical patients). In both groups, the highest incidence of CPRA occurred in patients managed by a combined treatment with both type of drugs (see Table 1). In the medical patient group exposed to both sedative and opioid treatment, the CPRA rate increased to 7.54 per 1000 admissions as compared to 2.08 per 1000 admissions in medical patients without this treatment. Surgical patients on both sedatives and opioids had an even higher CPRA rate of 9.59 per 1000 admissions vs. 5.30 for those who were exposed to neither of these medications.

**Table 1:** Incidence of CPRA in medical vs. surgical patients on sedatives, opioids, both sedatives and opioids, or neither sedatives nor opioids.

Variable	Medical Patients Only					Surgical Patients Only				
	All Medical Patients	Sedatives Only	Opioids Only	Opioids and Sedatives	No Opioids or Sedatives	All Surgical Patients	Sedatives Only	Opioids Only	Opioids and Sedatives	No Opioids or Sedatives
**Total Patients**	14,504,809	2,619,567	3,184,007	2,448,268	6,252,967	6,771,882	411,355	3,487,766	2,206,046	666,715
**% of Whole Group**	100	18.06	21.95	16.88	43.11	100	6.07	51.50	32.58	9.85
**Total # of CPRA**	54,745	9,489	13,775	18,451	13,030	41,809	3,832	13,282	21,147	3,548
**% of Whole Group**	100	17.33	25.16	33.70	23.80	100	9.17	31.77	50.58	8.49
**CPRA rate / 1000 admissions**	3.77	3.62	4.33	7.54	2.08	6.17	9.32	3.81	9.59	5.30

CPRA = cardiopulmonary or respiratory arrest.

### Demographic and clinical characteristics

The demographics of the surgical and medical in-hospital patients were similar (see S3 Table). The mean ages were 57.7 and 56.4 years old for the medical group and the surgical group, respectively. In the medical group, there was a higher representation of patients above 80 years old (18.2% vs. 10.6%) and patients of the black race (14.7% vs. 10.6%) compared to the surgical group. In general, medical patients were sicker than surgical patients, with a higher proportion of individuals with a higher Charlson Comorbidity Index (CCI >2 in 25.1% of medical patients vs. 17.4% of surgical patients) and more advanced severity of the illness according to All Patients Refined Diagnosis Related Groups classification (the “major” and “extreme” APR severity levels included 34.9% of medical patients vs. 24.5% of surgical patients). Medical patients were more commonly admitted on a non-elective basis (84.3% vs. 45.5%). The most common pathologies in both medical and surgical groups were hypertension, diabetes, and COPD.

### Multivariable analysis

To determine which risk factors are associated with the increased odds in development of CPRA, a multivariable logistics regression analysis was performed. In both the medical and surgical groups, the use of opioids and sedatives were found to be independent and significant predictors of the occurrence of CPRA (Table 2). In the medical group the use of opioids alone was associated with a higher risk of CPRA as compared to sedatives (adjusted OR of 2.24 [95% CI: 2.18–2.29] vs. 1.80 [95% CI: 1.75–1.85], respectively). In contrast, in the surgical group, sedatives alone conveyed a higher risk than opioids alone (adjusted OR of 1.58 [95% CI: 1.51–1.66] vs. 1.12 [95% CI: 1.07–1.16], respectively). In both groups, however, the highest risk of developing CPRA was associated with the patients receiving both opioids and sedatives (adjusted ORs of 3.83 [95% CI: 3.74–3.92] in medical patients and 2.34 [95% CI: 2.25–2.42] in surgical patients)(Table 2).

**Table 2:** Multivariable logistic regression analysis of the incidence of CPRA.

Patient Population	Variable		Sedatives Only	Opioids Only	Both Opioids and Sedatives	Neither Opioids nor Sedatives
**Medical Patients** **(c-statistic = 0.763)**	**With CPRA (n = 54,745)**	**n**	9,489	13,775	18,451	13,030
		**row %**	17.33	25.16	33.70	23.80
	**Without CPRA (n = 14,450,064)**	**n**	2,610,078	3,170,232	2,429,817	6,239,937
		**row %**	18.06	21.94	16.82	43.18
	**Adjusted Odds Ratio (95% CI)** ^**a**^		1.80 (1.75, 1.85)	2.24 (2.18, 2.29)	3.83 (3.74, 3.92)	ref.
	***P*-value**		<0.0001	<0.0001	<0.0001	
**Surgical Patients** **(c-statistic = 0.812)**	**With CPRA (n = 41,809)**	**n**	3,832	13,282	21,147	3,548
		**row %**	9.17	31.77	50.58	8.49
	**Without CPRA (n = 6,730,073)**	**n**	407,523	3,474,484	2,184,899	663,167
		**row %**	6.06	51.63	32.46	9.85
	**Adjusted Odds Ratio (95% CI)** ^**a**^		1.58 (1.51, 1.66)	1.12 (1.07, 1.16)	2.34 (2.25, 2.42)	ref.
	***P*-value**		<0.0001	<0.0001	<0.0001	

^a^ The adjusted OR (odds ratio) was estimated by a multivariable logistic regression analysis, which included opioid usage, age, gender, race, comorbidity conditions, admission type, and hospital characteristics (including region, bed size, rural vs. urban, and teaching vs. non-teaching hospital) for medical and surgical patients, respectively.

CI = confidence interval; CPRA = cardiopulmonary or respiratory arrest; ref. = reference.

The patient population on both opioids and sedatives was separately analyzed with a multivariable logistic regression analysis to determine which of the risk factors might contribute to CPRA in patients. A total of four groups were analyzed: medical and surgical patients that received either opioids and sedatives or neither of them (see S4 Table). Table 3 summarizes data on independent predictors that increase the risk of CPRA only in medical or surgical patients on opioids and sedatives. These specific risk factors were not present in patients taking neither of these drugs. As seen in Table 3, there were risk factors for CPRA that were unique to medical and surgical patient groups as well as risk factors common to both groups.

**Table 3:** Specific independent predictors of CPRA in medical vs. surgical patients on opioid and sedative therapy.

Medical Patients		Surgical Patients	
Predictors for Increased Risk of CPRA	Adjusted OR ^a^ (95% CI)	Predictors for Increased Risk of CPRA	Adjusted OR ^a^ (95% CI)
**Hispanic race** ^**b**^	1.31 (1.20, 1.42)	Male Gender ^c^	1.45 (1.41, 1.49)
**Mild liver disease**	1.25 (1.15, 1.36)	Age: 61–70 ^d^	1.41 (1.35, 1.48)
**Obesity**	1.12 (1.06, 1.17)	**Hispanic race** ^**b**^	1.32 (1.22, 1.42)
Peptic ulcer disease	1.10 (1.00, 1.20)	Cancer	1.29 (1.23, 1.36)
**COPD**	1.09 (1.06, 1.13)	Race–other ^**b**^	1.24 (1.19, 1.29)
		**Mild liver disease**	1.21 (1.08, 1.35)
		Age: 51–60 ^d^	1.20 (1.15, 1.26)
		**COPD**	1.16 (1.13, 1.20)
		Metastatic solid tumor	1.16 (1.08, 1.25)
		Urban—Hospital Location ^e^	1.16 (1.10, 1.22)
		**Obesity**	1.08 (1.04, 1.11)
		Diabetes	1.08 (1.04, 1.13)
		Sleep disorder	1.07 (1.01, 1.12)

^a^ The adjusted OR (odds ratio) estimation was based on a multivariable logistic regression analysis, which included opioid usage, age, gender, race, comorbidity condition, admission type, and hospital characteristics (including region, bed size, rural vs. urban, and teaching vs. non-teaching hospital). For reference, see a complete description of the models in S4 Table in the Supplemental Section.

^b^ “Race–other” includes patients who do not identify themselves as white race, black race, or Hispanic origin; reference group: white race

^c^ Reference group: female

^d^ Reference group: age 18–50

^e^ Reference group: rural hospital.

All the risk factors are shown are ranked by adjusted odds ratio (OR; *p*<0.05) shown in a decreasing order of the effect size. Bold font identifies factors similar between medical and surgical patients.

COPD = chronic obstructive pulmonary disease; CPRA = cardiopulmonary or respiratory arrest.

### Survival in patients with CPRA

Over 71% of CPRA cases led to death for medical patients as compared to 46% for surgical patients. For medical patients who developed CPRA, the odds ratio of surviving was only about one third of surgical CPRA patients (OR 0.343 [95% CI: 0.334–0.352]). A similar odds ratio of survival in medical vs. surgical patients was noted in patients on opioid and sedatives who developed CPRA (OR 0.346 [95% CI: 0.332–0.360]).

## Discussion

The main finding of the study was the demonstration that both medical and surgical patients had an elevated risk of in-hospital CPRA when treated with opioids and sedatives. Opioids and sedatives had an independent and additive effect on the risk of developing CPRA in each clinical group of patients (see Table 2). As compared to patients who received treatment with opioids only, those who received additional sedative medications had a twofold increase in the risk of developing CPRA (a 2.0- and. 1.7-times increase in risk in the surgical and the medical group, respectively; see Table 2). Medical and surgical patients treated with opioid and sedative therapy were shown to have the specific risk factors of CPRA, which were not present in patients taking neither of these drugs (see Table 3). The present study also confirmed a high mortality risk for patients who developed CPRA. In the United States and other developed countries, as much as 3.4% to 6% of in-hospital death is preventable [18]. OIRD represents one of the major causes of the preventable in-hospital death. In 2012, The Joint Commission issued a sentinel event alert on safe use of opioids in hospitals [7]. This document underscores the importance of considering an elevated risk of opioids in various groups of patients to avoid the potential for oversedation and respiratory depression. The knowledge of which patients require special attention is needed to improve the safe use of opioid and sedative therapy [19].

There were some similarities and differences in the characteristics between in-hospital medical and surgical patients in the presentation of the specific patterns of CPRA risk while being on opioid and sedative therapy. Surgical patients had a greater exposure to opioids and sedatives, a higher rate of CPRA, and a greater survival from CPRA than medical patients. In part, a better survival could be attributed to a better health status of the surgical patients. In our multivariable analysis for both medical and surgical groups, four factors came up as independent predictors increasing the risk of developing CPRA while on opioid and sedative therapy: Hispanic origin, obesity, mild liver disease, and COPD. We did not observe these associations in the related control groups of patients without opioid and sedative medications.

There is a paucity of data on the incidence of ICHA in different racial and ethnic populations [20]. In our study, patients of Hispanic origin on opioids and sedatives, in both medical and surgical groups, had a higher unadjusted incidence of CPRA (7.56 and 10.12 per 1000 admission, respectively) as compared to the patients of a white race (6.87 and 8.50 per 1000 admission, respectively). Patients of a black race had an even higher rate of CPRA than other ethnic groups (10.19 and 16.07 per 1000 medical and surgical admission, respectively). However, the black race as a risk-adjusted predictor of CPRA was significant across all groups and independent of the use of opioids and sedatives. The increased risk of the Hispanic population may be explained by differences in socioeconomic factors, individual characteristics, or elements of the health care system [20]. It has been reported that Hispanics have less exposure to opioids by having less prescription medication [21] and slightly less current illicit drug use [22] as compared to whites. Our study observation confirms that Hispanics were less exposed to in-hospital opioid and sedative therapy as compared to whites in both medical (13.5 vs. 18.0%) and surgical wards (26.9 vs. 34.7%). The reason this patient population is more vulnerable to CPRA with the opioid and sedative treatment needs further evaluation.

Obesity has long been an important public health priority [23]. It raises the risk for all-cause and cardiovascular mortality [24], and predisposes patients to post-anesthesia critical respiratory events [25]. Identification of obesity as a risk factor for obstructive sleep apnea (OSA) [26] became an important aspect in managing patients during the perioperative period and in those receiving opioids for pain control [27, 28]. In our study, however, obesity was a risk predictor for CPRA, independent of other important clinical confounders including a sleep disorder. Further predispositions in the obese patient population to the complications from opioid and sedative medications include a higher frequency of daily pain [29], an altered response of the endogenous opioid system [30], different pharmacokinetics of sedative and pain control medications [31], and obesity hypoventilation syndrome [32].

Liver disease may impair the metabolism and excretion of many opioid and sedative medications [33], thereby lowering the threshold for overdose. Child-Pugh and the model for end-stage liver disease (MELD) classifications of the severity of liver disease became useful clinical indices in predicting mortality for these patients and their perioperative outcomes [33, 34]. In our study, moderate to severe liver disease was a major comorbidity factor with the highest risk for developing in-hospital CPRA across all groups and independent of the use of opioids and sedatives. Interestingly, our results bring particular attention to the group of medical and surgical patients with the mild form of liver disease. They had no additional risk of developing in-hospital CPRA unless they received opioids and sedatives. A recent study of Veterans’ Health Administration population on opioid prescription [35] has found that pre-existing mild liver disease was associated with elevated risk for serious opioid-induced respiratory depression and overdose events.

COPD is one of the leading causes of mortality and a major contributor to hospital admission [36]. Drug-induced respiratory depression makes patients with preexisting respiratory disease and COPD susceptible to opioid overdose [37] in both outpatient [35] and in-hospital settings [38]. A recent Swedish study of COPD patients [39] revealed that benzodiazepines and higher dose opioids are associated with elevated mortality risks, especially when used concurrently. Our study has demonstrated that use of both opioids and sedatives increases the risk CPRA in both medical and surgical patients with COPD.

Peptic ulcer disease (PUD) was shown in this study to be associated with an increased risk of CPRA only for medical patients on opioids and sedatives. The nature of this association is unclear and might reflect a link between PUD and an elevated risk for gastrointestinal bleeding in critically ill patients on sedation protocols [40]. In a subpopulation of surgical patients treated with opioids and sedatives, PUD did not present itself as a specific risk predictor of CPRA.

In patients receiving opioids and sedatives, surgical patients appear to have a different pattern of risk factors for CPRA, compared to medical patients (see Table 3). This includes certain demographic factors, comorbidities, and hospital settings. Previously, it has been reported that male gender and older age have been associated with postsurgical opioid-related adverse events [41]. The same demographic factors were also implied as risk factors for OSA [38]. In our multivariable model adjusted for confounders, we demonstrated that male gender and older age are risk predictors of CPRA, independent of the presence of a sleep disorder. While elderly patients over 70 years old were at risk for CPRA in all patient groups, the surgical cohort incurs an elevated risk after 50 years of age. Another demographic risk factor specific for the surgical group of patients was a race other than white, black, or Hispanic origin. This finding is of interest and needs further investigation to determine other ethnic groups at risk; further elucidation of race is not available within the Premier database.

Surgical patients with cancer or a metastatic solid tumor exhibited a higher risk of CPRA when receiving opioid and sedative therapy. Management of cancer patients after surgery often results in increased use of opioid since many are tolerant from opioid treatment of chronic pain [42]. However, we found opioid tolerant postsurgical patients to have a decreased risk for CPRA (see chronic pain patients in S4 Table). This may be due to the induction of enzymes that metabolize opioids, increasing margin of safety. Thus, within surgical chronic pain patients, cancer patients may represent a distinct subgroup in their response to opioid and sedative therapy, which makes them prone to the increase CPRA risk. This could be explained by the increased complexity and length of oncologic surgical procedures and reconstructions [43], requiring longer duration of anesthesia and more challenging postoperative recovery.

Diabetes and diabetic autonomic neuropathy predispose patients to perioperative hemodynamic instability and cardiac arrest [44]. In our study, only the diabetic surgical patients on opioid and sedative medications exhibited a higher risk of developing CPRA. Evidence implicating OSA in the pathogenesis of insulin resistance, glucose intolerance, type 2 diabetes, and the metabolic syndrome is accumulating [45]. Some diabetic features including a reduced hypoxic-induced ventilator drive [46], impaired immune system responses [47], and gastroparesis might predispose these patients to adverse cardiorespiratory events after surgery. Opioid induced respiratory depression [28], immunosuppression [48], and slowing gastrointestinal motility, may further contribute to poor perioperative outcomes in this patient population. In addition, patients with diabetic neuropathic pain [49] may require higher analgesic doses.

In our study, sleep disorder was associated with an elevated risk for CPRA in the surgical group of patients on opioid and sedative therapy. Sleep-related breathing disorders, especially OSA, are known risk factors for opioid induced respiratory depression and CPRA in the perioperative environment [7, 8, 26–28]. Partial or complete periodic obstruction of the airway associated with OSA leads to hypoxia and hypercarbia episodes, as well as cardiovascular dysfunction. Recognizing the major morbidity and mortality risks in OSA patients on opioids, recent professional guidelines [27, 28, 38] consider early recognition of patients at risk and the use of more vigilant monitoring of respiratory function and sedation levels for safe perioperative management of these patients.

We found that patients in urban hospitals had a higher risk of developing of CPRA when placed on opioid and sedative therapy. In outpatient settings, mortality as a result of opioid overdose was reported higher in rural areas [50]. A recent study of critical access hospitals [51], which serve mainly rural areas, has demonstrated that for the most common general surgeries, the risk for developing a major complication after surgery, such as pulmonary failure and myocardial infarction, was lower at rural hospitals, as compared to hospitals serving urban areas. In light of this data, our findings revealing the role of opioid and sedative medications in the perioperative period may provide some insight to the differences in cardiorespiratory outcomes between surgical patients in urban and rural areas. Further understanding the differences in hospital care between different hospitals could help develop targeted strategies of care in patients under the risk of IHCA [52, 53].

As a retrospective administrative database analysis, this study does not establish the causality or the temporality of the events but rather determines statistically significant associations between the clinical factors and outcomes. Drawing clinical inferences from an administrative database, has known limitations [14, 41]. Administrative databases capture only billing claims. It is possible that some clinical data might not be available. Data, such as the chronology of events, family history, severity of most disorders, laboratory data, vital signs, medication dosages, and “Do not resuscitate” status, are not listed in the administrative database. These additional features would provide more insight to identifications of specific risks associated with in-hospital opioid and sedative treatment. However, in this study, the important information about cardiopulmonary arrest is not likely to be overlooked. As shown in a study of Makadia and Ryan [54], the Premier database can be successfully used for the assessment of the quality of hospital care where the quality metrics of the study rely on information contained within the inpatient encounter. While many medications have sedative properties, we have limited our analysis to opioids and sedatives commonly used on hospital wards and previously shown to be associated with the development of inpatient CPRA [14]. It is unknown how the inclusion (or exclusion) of specific medications from the analysis would impact the findings. Our study has strength related to the exceptionally large number of the patients analyzed. The results are applicable to the diverse set of hospital conditions. Since the Premier database includes privately and publically insured, as well as, uninsured patients, it is more representative of the national patient population compared to data from Medicare or private insurance databases.

In conclusion, opioids and sedatives are independent and additive predictors of CPRA in both medical and surgical in-hospital patients. Using the risk stratification identified by this study, judicious use of the combination of opioids with sedative medications, opioids sparing analgesic techniques, and more precise monitoring of selected high-risk patients may help prevent these catastrophic events.

## Supporting information

S1 Table Exclusionary ICD-9-CM codes. (DOCX)

S2 Table Types of medications included in the study. (DOCX)

S3 Table Demographic, clinical, and provider characteristics in studied medical and surgical inpatients. (DOCX)

S4 Table Adjusted odds ratios for CPRA for medical and surgical patients on opioids and sedatives or neither of them. (DOCX)
