# Supplementary material for: Risk factors for cardiopulmonary and respiratory arrest in medical and surgical hospital patients on opioid analgesics and sedatives
Source: PLoS One. 2018 Mar 22;13(3):e0194553. doi: 10.1371/journal.pone.0194553 (PMC5864099; doi:10.1371/journal.pone.0194553)
Supplement: S3 Table — (DOCX) [file pone.0194553.s003.docx]

**S3 Table. Demographic, Clinical, and Provider Characteristics in Studied Medical and Surgical Inpatients.**

| **Characteristic** | **Medical patients (n=14,504,809)** | | **Surgical patients (n=6,771,882)** | |
| --- | --- | --- | --- | --- |
|  | **n** | **col %** | **n** | **col %** |
| **Age** |  | |  | |
| Overall | 57.7 ± 21.1 | | 56.4 ± 18.9 | |
| 80+ | 2,641,605 | 18.21 | 714,616 | 10.55 |
| 71 - 80 | 2,250,599 | 15.52 | 1,101,386 | 16.26 |
| 61 - 70 | 2,121,651 | 14.63 | 1,320,669 | 19.50 |
| 51 - 60 | 2,080,315 | 14.34 | 1,153,210 | 17.03 |
| 18 - 50 | 5,410,639 | 37.30 | 2,482,001 | 36.65 |
| **CCI** |  | |  | |
| Overall | 1.68 ± 2.13 | | 1.29 ± 1.95 | |
| CCI > 2 | 3,646,282 | 25.14 | 1,180,677 | 17.43 |
| CCI = 2 | 2,046,603 | 14.11 | 885,823 | 13.08 |
| CCI = 1 | 3,049,907 | 21.03 | 1,356,952 | 20.04 |
| CCI = 0 | 5,762,017 | 39.72 | 3,348,430 | 49.45 |
| **APR severity of illness** |  |  |  |  |
| Extreme | 872,428 | 6.01 | 431,259 | 6.37 |
| Major | 4,189,880 | 28.89 | 1,230,625 | 18.17 |
| Moderate | 5,870,253 | 40.47 | 2,450,649 | 36.19 |
| Minor | 3,572,248 | 24.63 | 2,659,349 | 39.27 |
| **Gender** |  |  |  |  |
| Male | 5,780,176 | 39.85 | 2,687,436 | 39.69 |
| Female | 8,724,633 | 60.15 | 4,084,446 | 60.31 |
| **Race** |  |  |  |  |
| White | 9,243,370 | 63.73 | 4,602,982 | 67.97 |
| Black | 2,133,275 | 14.71 | 719,763 | 10.63 |
| Hispanic | 611,965 | 4.22 | 271,363 | 4.01 |
| Other | 2,516,199 | 17.35 | 1,177,774 | 17.39 |
| **MDC of 14 (Pregnancy, Childbirth, and Puerperium)** | 1,901,263 | 13.11 | 962,509 | 14.21 |
| **With Comorbidity Condition** |  |  |  |  |
| Hypertension | 7,569,039 | 52.18 | 3,364,075 | 49.68 |
| Diabetes (with or without sequelae) | 3,702,817 | 25.53 | 1,425,055 | 21.04 |
| COPD | 3,166,310 | 21.83 | 1,033,427 | 15.26 |
| Congestive heart failure | 2,153,479 | 14.85 | 544,671 | 8.04 |
| Chronic renal failure | 2,113,501 | 14.57 | 600,265 | 8.86 |
| History of Smoking | 1,376,509 | 9.49 | 734,986 | 10.85 |
| Obesity | 1,437,414 | 9.91 | 929,508 | 13.73 |
| Cardiovascular disease | 1,222,008 | 8.42 | 317,473 | 4.69 |
| Myocardial infarction | 1,051,177 | 7.25 | 634,390 | 9.37 |
| Cancer | 1,003,242 | 6.92 | 597,855 | 8.83 |
| Sleep disorder | 712,648 | 4.91 | 410,285 | 6.06 |
| Peripheral Vascular Disease | 655,912 | 4.52 | 402,712 | 5.95 |
| History of opioids use/Chronic pain | 496,759 | 3.42 | 136,610 | 2.02 |
| Metastatic solid tumor | 458,879 | 3.16 | 197,826 | 2.92 |
| Rheumatoid arthritis | 374,177 | 2.58 | 153,258 | 2.26 |
| Mild liver disease | 304,702 | 2.10 | 48,893 | 0.72 |
| Peptic ulcer disease | 258,885 | 1.78 | 62,488 | 0.92 |
| Moderate-Severe Liver disease | 200,828 | 1.38 | 23,474 | 0.35 |
| Paralysis | 199,249 | 1.37 | 50,035 | 0.74 |
| Dementia | 147,701 | 1.02 | 20,866 | 0.31 |
| AIDS | 72,968 | 0.50 | 8,952 | 0.13 |
| **Admission type** |  |  |  |  |
| Non-elective | 12,227,858 | 84.30 | 3,084,173 | 45.54 |
| Elective | 2,276,951 | 15.70 | 3,687,709 | 54.46 |
| **Region** |  |  |  |  |
| South | 5,970,540 | 41.16 | 2,824,793 | 41.71 |
| Northeast | 3,060,528 | 21.10 | 1,330,156 | 19.64 |
| Midwest | 2,963,593 | 20.43 | 1,290,996 | 19.06 |
| West | 2,510,148 | 17.31 | 1,325,937 | 19.58 |
| **Teaching hospital** | 5,596,982 | 38.59 | 2,856,813 | 42.19 |
| **Hospital Bed size** |  |  |  |  |
| > 500 | 4,051,317 | 27.93 | 2,206,687 | 32.59 |
| 250 - 500 | 6,813,870 | 46.98 | 3,174,801 | 46.88 |
| < 250 | 3,639,622 | 25.09 | 1,390,394 | 20.53 |
| **Hospital Location** |  |  |  |  |
| Urban | 12,777,854 | 88.09 | 6,113,106 | 90.27 |
| Rural | 1,726,955 | 11.91 | 658,776 | 9.73 |

Values presented as Mean ± SD. AIDS = acquired immune deficiency syndrome; APR = All Patient Refined Severity of Illness score; CCI = Charlson Comorbidity Index; col = column; COPD = chronic obstructive pulmonary disease.
