# Supplementary material for: Risk factors for cardiopulmonary and respiratory arrest in medical and surgical hospital patients on opioid analgesics and sedatives
Source: PLoS One. 2018 Mar 22;13(3):e0194553. doi: 10.1371/journal.pone.0194553 (PMC5864099; doi:10.1371/journal.pone.0194553)
Supplement: S4 Table — (DOCX) [file pone.0194553.s004.docx]

**S4 Table. Adjusted Odds Ratios for CPRA for Medical Patients on Opioids and Sedatives or Neither of Them.**

| **Factor** | **Medical Patients with both Opioids and Sedatives (n=2,448,268; c-statistic= 0.76)** | | **Medical Patients with neither Opioids nor Sedatives (n=6,252,967; c-statistic= 0.71)** | | **Surgical Patients with both Opioids and Sedatives (n=2,206,046; c-statistic= 0.86)** | | **Surgical Patients with neither Opioids nor Sedatives (n=666,715; c-statistic= 0.81)** | |
| --- | --- | --- | --- | --- | --- | --- | --- | --- |
|  | **Adjusted OR*** | **P-value** | **Adjusted OR*** | **P-value** | **Adjusted OR*** | **P-value** | **Adjusted OR*** | **P-value** |
| **Age** |  |  |  |  |  |  |  |  |
| 80+ | 2.45 (2.33, 2.59) | <0.0001 | 1.95 (1.83, 2.08) | <0.0001 | 1.74 (1.65, 1.83) | <0.0001 | 1.40 (1.24, 1.59) | <0.0001 |
| 71 - 80 | 2.26 (2.15, 2.38) | <0.0001 | 1.84 (1.73, 1.97) | <0.0001 | 1.67 (1.60, 1.76) | <0.0001 | 1.30 (1.16, 1.47) | <0.0001 |
| 61 - 70 | 1.83 (1.74, 1.92) | <0.0001 | 1.50 (1.40, 1.61) | <0.0001 | 1.41 (1.35, 1.48) | <0.0001 | 1.10 (0.97, 1.24) | 0.1309 |
| 51 - 60 | 1.37 (1.31, 1.44) | <0.0001 | 1.34 (1.24, 1.44) | <0.0001 | 1.20 (1.15, 1.26) | <0.0001 | 1.00 (0.88, 1.14) | 0.9793 |
| 18 - 50 | Ref. | | Ref. | | Ref. | | Ref. | |
| **Gender** |  |  |  |  |  |  |  |  |
| Male | 1.50 (1.46, 1.55) | <0.0001 | 1.26 (1.21, 1.30) | <0.0001 | 1.45 (1.41, 1.49) | <0.0001 | 0.97 (0.90, 1.04) | 0.3722 |
| Pregnant Female | 0.20 (0.15, 0.26) | <0.0001 | 0.10 (0.09, 0.12) | <0.0001 | 0.20 (0.16, 0.23) | <0.0001 | 0.12 (0.08, 0.17) | <0.0001 |
| Non-Pregnant Female | Ref. | | Ref. | | Ref. | | Ref. | |
| **Race** |  |  |  |  |  |  |  |  |
| Black | 1.76 (1.69, 1.83) | <0.0001 | 1.79 (1.71, 1.88) | <0.0001 | 1.94 (1.86, 2.02) | <0.0001 | 1.38 (1.24, 1.54) | <0.0001 |
| Hispanic | 1.31 (1.20, 1.42) | <0.0001 | 1.08 (0.98, 1.19) | 0.1188 | 1.32 (1.22, 1.42) | <0.0001 | 1.16 (0.96, 1.39) | 0.1157 |
| Other | 1.25 (1.20, 1.31) | <0.0001 | 1.24 (1.18, 1.30) | <0.0001 | 1.24 (1.19, 1.29) | <0.0001 | 0.96 (0.88, 1.05) | 0.4023 |
| White | Ref. | | Ref. | | Ref. | | Ref. | |
| **Admission type** |  |  |  |  |  |  |  |  |
| Non-elective | 1.27 (1.21, 1.35) | <0.0001 | 1.12 (1.05, 1.19) | 0.0003 | 2.12 (2.05, 2.19) | <0.0001 | 2.35 (2.16, 2.55) | <0.0001 |
| Elective | Ref. | | Ref. | | Ref. | | Ref. | |
| **Myocardial Infarction** |  |  |  |  |  |  |  |  |
| Yes | 1.99 (1.92, 2.07) | <0.0001 | 2.35 (2.25, 2.46) | <0.0001 | 2.20 (2.13, 2.27) | <0.0001 | 2.15 (2.00, 2.31) | <0.0001 |
| No | Ref. | | Ref. | | Ref. | | Ref. | |
| **Congestive Heart Failure** |  |  |  |  |  |  |  |  |
| Yes | 2.24 (2.16, 2.32) | <0.0001 | 1.65 (1.59, 1.72) | <0.0001 | 2.57 (2.49, 2.66) | <0.0001 | 1.76 (1.63, 1.91) | <0.0001 |
| No | Ref. | | Ref. | | Ref. | | Ref. | |
| **Dementia** |  |  |  |  |  |  |  |  |
| Yes | 0.99 (0.87, 1.12) | 0.8513 | 1.11 (0.89, 1.16) | 0.8163 | 0.74 (0.62, 0.88) | 0.0009 | 0.84 (0.57, 1.26) | 0.4042 |
| No | Ref. | | Ref. | | Ref. | | Ref. | |
| **COPD** |  |  |  |  |  |  |  |  |
| Yes | 1.09 (1.06, 1.13) | <0.0001 | 0.91 (0.87, 0.95) | <0.0001 | 1.16 (1.13, 1.20) | <0.0001 | 1.06 (0.97, 1.15) | 0.2211 |
| No | Ref. | | Ref. | | Ref. | | Ref. | |
| **Rheumatoid Arthritis** |  |  |  |  |  |  |  |  |
| Yes | 1.11 (0.94, 1.10) | 0.631 | 1.01 (0.91, 1.13) | 0.8158 | 1.00 (0.92, 1.08) | 0.9398 | 0.97 (0.77, 1.21) | 0.7678 |
| No | Ref. | | Ref. | | Ref. | | Ref. | |
| **Peptic Ulcer Disease** |  |  |  |  |  |  |  |  |
| Yes | 1.10 (1.00, 1.20) | 0.0394 | 1.12 (0.99, 1.27) | 0.0775 | 1.85 (1.72, 1.99) | <0.0001 | 1.99 (1.57, 2.52) | <0.0001 |
| No | Ref. | | Ref. | | Ref. | | Ref. | |
| **Paralysis** |  |  |  |  |  |  |  |  |
| Yes | 1.31 (1.20, 1.44) | <0.0001 | 1.44 (1.28, 1.63) | <0.0001 | 1.55 (1.42, 1.69) | <0.0001 | 1.71 (1.32, 2.21) | <0.0001 |
| No | Ref. | | Ref. | | Ref. | | Ref. | |
| **Chronic Renal Failure** |  |  |  |  |  |  |  |  |
| Yes | 0.80 (1.74, 1.86) | <0.0001 | 1.63 (1.56, 1.70) | <0.0001 | 1.92 (1.86, 1.99) | <0.0001 | 1.47 (1.35, 1.60) | <0.0001 |
| No | Ref. | | Ref. | | Ref. | | Ref. | |
| **Cancer** |  |  |  |  |  |  |  |  |
| Yes | 1.40 (1.33, 1.47) | <0.0001 | 1.37 (1.28, 1.46) | <0.0001 | 1.29 (1.23, 1.36) | <0.0001 | 1.06 (0.92, 1.22) | 0.438 |
| No | Ref. | | Ref. | | Ref. | | Ref. | |
| **Metastatic Solid Tumor** |  |  |  |  |  |  |  |  |
| Yes | 1.21 (1.14, 1.29) | <0.0001 | 1.51 (1.36, 1.67) | <0.0001 | 1.16 (1.08, 1.25) | <0.0001 | 1.05 (0.83, 1.33) | 0.6984 |
| No | Ref. | | Ref. | | Ref. | | Ref. | |
| **AIDS** |  |  |  |  |  |  |  |  |
| Yes | 2.04 (1.80, 2.31) | <0.0001 | 1.44 (1.14, 1.81) | 0.0022 | 1.59 (1.29, 1.96) | <0.0001 | 2.01 (1.07, 3.78) | 0.0306 |
| No | Ref. | | Ref. | | Ref. | | Ref. | |
| **Obesity** |  |  |  |  |  |  |  |  |
| Yes | 1.12 (1.06, 1.17) | <0.0001 | 0.93 (0.87, 0.99) | 0.0208 | 1.08 (1.04, 1.13) | <0.0001 | 1.07 (0.96, 1.18) | 0.2356 |
| No | Ref. | | Ref. | | Ref. | | Ref. | |
| **Diabetes (with or without Sequelae)** |  |  |  |  |  |  |  |  |
| Yes | 1.03 (0.99, 1.06) | 0.1154 | 1.03 (0.99, 1.07) | 0.1782 | 1.08 (1.04, 1.11) | <0.0001 | 0.94 (0.87, 1.01) | 0.0981 |
| No | Ref. | | Ref. | | Ref. | | Ref. | |
| **Hypertension** |  |  |  |  |  |  |  |  |
| Yes | 0.82 (0.79, 0.85) | <0.0001 | 0.64 (0.61, 0.66) | <0.0001 | 0.82 (0.79, 0.85) | <0.0001 | 0.69 (0.64, 0.75) | <0.0001 |
| No | Ref. | | Ref. | | Ref. | | Ref. | |
| **Peripheral Vascular Disease** |  |  |  |  |  |  |  |  |
| Yes | 1.19 (1.13, 1.25) | <0.0001 | 1.34 (1.26, 1.42) | <0.0001 | 1.25 (1.20, 1.30) | <0.0001 | 1.52 (1.37, 1.68) | <0.0001 |
| No | Ref. | | Ref. | | Ref. | | Ref. | |
| **Cardiovascular Disease** |  |  |  |  |  |  |  |  |
| Yes | 1.43 (1.36, 1.50) | <0.0001 | 1.06 (1.01, 1.12) | 0.0328 | 1.60 (1.53, 1.68) | <0.0001 | 1.33 (1.19, 1.49) | <0.0001 |
| No | Ref. | | Ref. | | Ref. | | Ref. | |
| **Mild Liver Disease** |  |  |  |  |  |  |  |  |
| Yes | 1.25 (1.15, 1.36) | <0.0001 | 1.09 (0.95, 1.26) | 0.212 | 1.21 (1.08, 1.35) | 0.0007 | 1.43 (1.00, 2.04) | 0.05 |
| No | Ref. | | Ref. | | Ref. | | Ref. | |
| **Moderate-Severe Liver Disease** |  |  |  |  |  |  |  |  |
| Yes | 2.51 (2.31, 2.73) | <0.0001 | 2.61 (2.26, 3.02) | <0.0001 | 2.64 (2.35, 2.98) | <0.0001 | 2.60 (1.74, 3.88) | <0.0001 |
| No | Ref. | | Ref. | | Ref. | | Ref. | |
| **History of Opioids Use/Chronic pain** |  |  |  |  |  |  |  |  |
| Yes | 0.58 (0.53, 0.62) | <0.0001 | 0.84 (0.73, 0.97) | 0.0198 | 0.74 (0.68, 0.81) | <0.0001 | 0.82 (0.60, 1.11) | 0.2019 |
| No | Ref. | | Ref. | | Ref. | | Ref. | |
| **Sleep Disorder** |  |  |  |  |  |  |  |  |
| Yes | 1.02 (0.96, 1.08) | 0.5964 | 0.96 (0.89, 1.04) | 0.3471 | 1.07 (1.01, 1.12) | 0.0124 | 1.01 (0.87, 1.17) | 0.9121 |
| No | Ref. | | Ref. | | Ref. | | Ref. | |
| **History of Smoking** |  |  |  |  |  |  |  |  |
| Yes | 1.02 (0.98, 1.06) | 0.3978 | 0.82 (0.78, 0.87) | <0.0001 | 0.92 (0.88, 0.96) | <0.0001 | 0.76 (0.69, 0.85) | <0.0001 |
| No | Ref. | | Ref. | | Ref. | | Ref. | |
| **Region** |  |  |  |  |  |  |  |  |
| South | 1.01 (0.97, 1.05) | 0.803 | 0.96 (0.91, 1.01) | 0.1294 | 0.89 (0.86, 0.93) | <0.0001 | 0.78 (0.70, 0.87) | <0.0001 |
| Northeast | 0.95 (0.90, 1.01) | 0.0799 | 0.68 (0.64, 0.72) | <0.0001 | 0.93 (0.88, 0.97) | 0.0024 | 0.52 (0.47, 0.59) | <0.0001 |
| Midwest | 0.81 (0.77, 0.86) | <0.0001 | 0.87 (0.82, 0.92) | <0.0001 | 0.85 (0.81, 0.89) | <0.0001 | 0.83 (0.74, 0.92) | 0.0007 |
| West | Ref. | | Ref. | | Ref. | | Ref. | |
| **Teaching Hospital** |  |  |  |  |  |  |  |  |
| Yes | 1.10 (1.06, 1.14) | <0.0001 | 1.18 (1.13, 1.23) | <0.0001 | 1.10 (1.06, 1.13) | <0.0001 | 1.14 (1.05, 1.23) | 0.0012 |
| No | Ref. | | Ref. | | Ref. | | Ref. | |
| **Hospital Bed Size** |  |  |  |  |  |  |  |  |
| > 500 | 0.97 (0.92, 1.01) | 0.1549 | 1.01 (0.95, 1.06) | 0.8301 | 1.07 (1.02, 1.12) | 0.0043 | 1.35 (1.19, 1.52) | <0.0001 |
| 250 - 500 | 1.09 (1.05, 1.13) | <0.0001 | 1.12 (1.07, 1.17) | <0.0001 | 1.15 (1.10, 1.19) | <0.0001 | 1.19 (1.07, 1.32) | 0.0014 |
| < 250 | Ref. | | Ref. | | Ref. | | Ref. | |
| **Hospital Location** |  |  |  |  |  |  |  |  |
| Urban | 0.96 (0.92, 1.01) | 0.1074 | 0.99 (0.94, 1.05) | 0.7118 | 1.16 (1.10, 1.22) | <0.0001 | 0.89 (0.79, 1.00) | 0.06 |
| Rural | Ref. | | Ref. | | Ref. | | Ref. | |

* The adjusted OR (odds ratio) was estimated by a multivariable logistic regression analysis, which included opioid usage, age, gender, race, comorbidity conditions, admission type, and hospital characteristics (including region, bed size, rural vs. urban, and teaching vs. non-teaching hospital). CPRA = cardiopulmonary or respiratory arrest; OR = odds ratio.
